# Mapping the subgingival HerBiome and HisBiome over the human healthspan

**DOI:** 10.1002/jper.70098

**Published:** 2026-03-23

**Authors:** Rahul Nikam, Kazune Pax, Michelle Lee‐Scott Beverly, Purnima S. Kumar

**Affiliations:** ^1^ Department of Periodontics and Oral Medicine School of Dentistry University of Michigan Ann Arbor Michigan USA; ^2^ Division of Oral Biology College of Dentistry The Ohio State University Columbus Ohio USA; ^3^ West Chester Center for Dentistry Cincinnati Ohio USA

**Keywords:** age, DNA sequence analysis, meta‐taxonomics, oral microbiome, sex, subgingival

## Abstract

**Background:**

Understanding the intricate relationship between sex, age, and the oral microbiome is crucial for deciphering the onset and progression of numerous age‐related oral and systemic diseases.

**Methods:**

Subgingival plaque was collected from 781 periodontally and systemically healthy females and 160 males spanning 0 to 80 years. 16S amplicon sequencing was performed. 80 million sequences were annotated and analyzed through the QIIME pipeline, principal components analysis (PCA) used for dimensionality reduction, LefSe to identify driver species, beta dispersion to measure inter‐subject variability, and machine learning algorithm (*RandomForest* package in R [RF]) to validate the results. Causal mediation models were implemented to investigate the influence of aging on the male and female microbiomes.

**Results:**

PCA demonstrated significant class separation based on sex (*p <* 0.001, permutational multivariate analysis of variance [PERMANOVA]). Males demonstrated higher alpha diversity (*p <* 0.001, Wilcoxon signed‐rank test of the Shannon diversity index), but also higher inter‐subject heterogeneity *p <* 0.001, ANOVA). RF identified males with 0.99 sensitivity, 0.15 specificity, and accuracy of 85%. Age exerted an almost complete mediation effect, with significant differences in the trajectory and pattern of aging between males and females. Females > 30 demonstrated a lower microbial diversity (*p <* 0.001) and higher levels of *Fusobacterium nucleatum* (*p <* 0.001), while the male microbiome remained highly personalized throughout the lifespan, without defined patterns of aging.

**Conclusions:**

Sex and age interact to influence the subgingival microbiome. These findings might explain differing disease susceptibilities in either sex, as well as informing personalized prevention and intervention based on age and sex. Further studies using granular ‐omics approaches are needed to advance our knowledge.

**Plain language summary:**

Periodontal (gum) diseases are caused by a breakdown in the intricate balance between bacteria that live under the gumline and the local immune response. Since periodontal diseases have been reported to be more common in men than in women, we set out to investigate whether these bacterial communities are intrinsically different between the 2 sexes, and whether these differences are sustained over the lifespan. Using deep‐sequencing technology to analyze the microbiomes of 941 individuals, we discovered that sex at birth is indeed a determining factor in the types of bacteria that live under the gums. Aging trajectories and patterns also differ between men and women, with women demonstrating a distinct shift after 30 years of age, and men showing no definite age‐based change. These findings have important implications for the cause of periodontitis in either sex, as well as the potential to personalize therapy based on age and sex.

## 1 INTRODUCTION

Periodontitis, one of the leading causes of tooth loss, is the sixth most common disease to affect humans; with an estimated 11% of the world's population suffering from severe forms of this disease.^1^ While the local consequences of periodontitis, that is, tooth mobility and loss, can have debilitating consequences with respect to speech, gustation, and nutrition,^2^ untreated periodontitis also has major implications for exacerbation of several systemic diseases.^3^, ^4^ Moreover, this disease imposes a significant economic burden on healthcare,^5^ with an annual loss of $154.06 billion in the United States and €158.64 billion in Europe.^6^ Therefore, risk‐based stratification of populations is key not only to establish personalized treatment paradigms, but also for effective healthcare delivery.

The literature is replete with studies demonstrating that the risk for periodontal diseases is not the same for all individuals.^2^, ^7^, ^8^ For example, the incidence and prevalence of periodontitis is reported to be higher among certain ethnic groups, after controlling for socio‐economic status and access to care.^9^ Similarly, it is now becoming increasingly apparent that sex is an independent variable in explaining the predisposition and susceptibility to periodontitis. Males have been reported to have a higher prevalence of periodontitis,^7^, ^8^, ^10^, ^11^ while hormonal fluctuations in females of reproductive age have been associated with higher incidence of gingival inflammation.^12^, ^13^, ^14^, ^15^

While many population‐based studies have implicated poorer home care regimens and oral hygiene in males as reasons for the higher prevalence,^16^ recent studies point to sexual dimorphisms in inflammatory cells and pathways as explanatory variables for these varying predispositions.^17^, ^18^ It is well established that periodontal diseases are initiated by a breakdown in host‐bacterial mutualism, and that microbial dysbiosis plays a causal role in disease etiology. Yet, sex‐based divergence of the subgingival microbiome has been entirely overlooked as a possible driver of these varying disease susceptibilities.

Emerging studies from the gut microbiome have demonstrated the existence of sex‐based differences in human‐to‐bacteria cell ratios (1:1.3 in men and 1:2.2 in women)^19^ as well as in community composition,^20^ giving rise to the terms “microsexome” or “microgenderome” to describe these sex‐specific biomes.^21^ These studies have expanded our understanding of the role of the microbiome in the etiopathogenesis of gut diseases and provide a roadmap for similar explorations with respect to the oral microbiome.

Recent studies have identified sex‐specific species enrichment in periodontitis‐associated subgingival biofilms.^22^ In parallel, our research group has identified the existence of sexual dimorphism in the oral microbiome within the first month of life (manuscript in revision, *International Society for Microbial Ecology Journal* [ISMEJ]), suggesting that the differences observed in the disease‐associated microbiomes of adults might be established much earlier in life, and prior to the onset of disease. However, the microbiome shifts compositionally in parallel with transitions from the pre‐dentate to the primary, mixed, and permanent dentition states.^23^ Hence, we do not know whether early‐life sexual dimorphism persists into adulthood.

Therefore, we used a deep sequencing meta‐taxonomic approach to investigate whether sex‐specific differences exist in microbiome composition, identify the age at which these differences become apparent, determine species responsible for these discrepancies, and explore how age influences microbial variations. To the best of our knowledge, we present the first available evidence on the interaction between age and sex on the subgingival microbiome in periodontally and systemically healthy individuals.

## 2 MATERIALS AND METHODS

### 2.1 Study approval

Approval for this study was obtained from the Office of Responsible Research Practices at The Ohio State University (Institutional Review Board [IRB] protocol numbers 2016H0155, 2018H0271, 2016H0199, and 2014H0062) and the study was conducted between 2016 and 2022 in accordance with the Helsinki Declaration as revised in 2013. Written informed consent was received from all subjects prior to participation.

### 2.2 Subject selection and recruitment

This study was a meta‐analysis of 4 datasets. 16S amplicon sequences (V1–V3 region) and metadata were available for analysis from 781 females and 160 males who satisfied the following inclusion and exclusion criteria. Inclusion criteria were individuals between 0 and 90 years of age, never smokers (< 100 cigarettes during the lifetime and not currently smoking) and non‐vapers (never used any electronic nicotine delivery system), periodontally healthy (attachment loss ≤ 1, no site with probe depths (PDs) > 4 mm, < 3 sites with PD of 4 mm, bleeding index (BOP) ≤ 20%), and systemically healthy (American Society of Anesthesiologists [ASA] I or II). Exclusion criteria included current pregnancy, antibiotic therapy or professional oral surgical or prophylactic procedures within the preceding 3 months, controlled or uncontrolled diabetes, and fewer than 20 teeth in the dentition.

### 2.3 Sample collection

Samples had been collected and pooled from mesial sites of 15 maxillary and mandibular single‐rooted teeth. Sites to be sampled had been isolated with cotton rolls, supragingival plaque carefully removed with scalers and air dried. Subgingival plaque was then collected using sterile endodontic paper‐points (Caulk‐Dentsply, Milford, DE, USA). Samples were placed in 1.5‐mL microcentrifuge tubes and frozen at −20°C until further analysis.

### 2.4 DNA isolation and sequencing

Bacterial DNA was isolated from plaque samples using a Qiagen DNA Miniamp kit (USA). Samples were incubated in lysozyme (Thermo Fisher Scientific, USA) for 90 minutes, and DNA was quantified with a Qubit fluorometer (Thermo Fisher Scientific, Waltham, MA, USA). The V1–V3 (spanning *Escherichia coli* 16S gene regions 8–27 and 519–536) sequenced as previously described^24^ on the MiSeq system (Illumina). The primers used for sequencing have been previously described (Kumar et al., 2011).^24^ Two of the sequence datasets have been uploaded to the Sequence Read Archive of the National Center for Biotechnology Information (NCBI) under the project numbers: PRJNA670727, PRJNA598720. The other 2 will be made available to researchers upon request and completion of data transfer agreements.

### 2.5 Bioinformatics and statistical analysis

**FIGURE 1:**
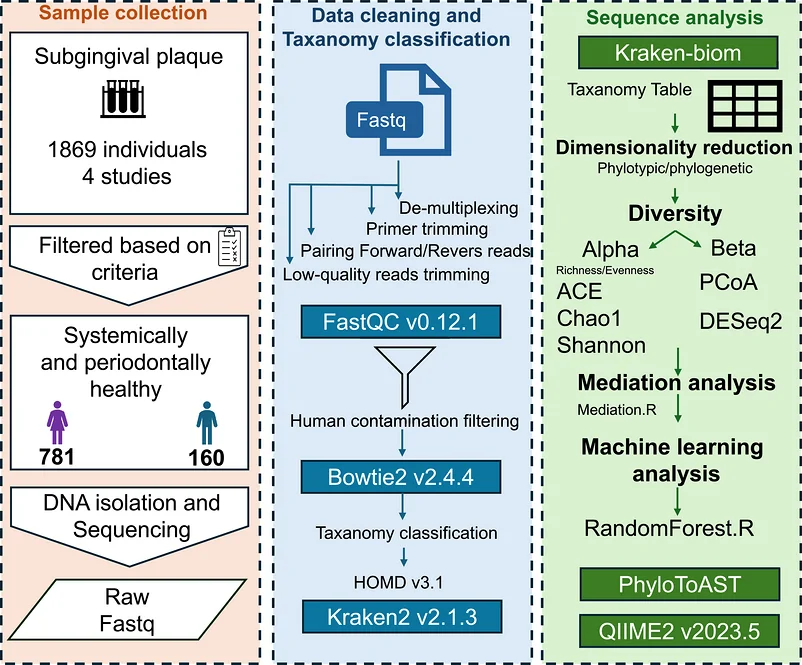
Overall pipeline of the analysis.

The quality of raw sequences was assessed with FastQC (v0.12.1), and low‐quality reads were trimmed using Sickle,^25^ and human contamination was filtered out using Bowtie2^26^ (v2.4.4). Reads were matched to target organisms using Kraken2^27^, ^28^ for taxonomic profiling with the Human Oral Microbiome Database^22^ (v3.1) as reference, and abundance matrix was then created from the Kraken reports using the Kraken‐Biom tool. Species richness was estimated using ACE and Chao indices, and alpha diversity using Shannon Diversity Index. Significance of group‐wise differences was tested using the Wilcoxon rank‐sum test. Principal coordinate analysis (PCoA) was used for dimensionality reduction and visualization. The significance of group‐wise clustering in the principal component analysis (PCA) was interrogated with a permutational multivariate analysis of variance (PERMANOVA) using the R package vegan.^29^ Species‐level operational taxonomic units (s‐OTUs) that mediated the differences between groups were identified using DESeq2.^30^ QIIME 2 (v2023.5)^31^ and PhyloToAST^32^ were used for bioinformatic analyses. Inter‐subject variability in microbial composition was estimated using beta dispersion^33^ of Bray–Curtis Dissimilarity Index. Causal mediation models were implemented using the mediate function in the “*mediation*” package in R.^34^, ^35^ We tested for interactions between sex as an explanatory variable and age as a mediator in models with beta diversity as the outcome. The ability of species to discriminate between groups was examined using a machine‐learning algorithm (*RandomForest* package in R^36^). Two‐thirds of the dataset was used to train the classifier, which was tested on the remaining data (1000 trees/10‐fold cross‐validation). A class weight balance was used to correct for the imbalance between the number of males and females in the study. For all iterations of the test a “confusion table” was created for each of the exposures based on the number of correctly classified and misclassified samples; and these data were used to compute sensitivity and specificity. The robustness of the classifier was evaluated using receiver operating characteristic (ROC) curves (*ROCR* package in R^37^). The pipeline used in this study is illustrated in Figure 1.

## 3 RESULTS

We obtained 8,60,06,639 classifiable sequences from the subgingival plaque of 781 systemically and periodontally healthy females and 160 males between 0 and 80 years of age to answer the following questions: (i) Is there evidence to support a subgingival microsexome? (ii) Which species are responsible for these differences? (iii) How does age influence these differences? (iv) At what age do these differences become apparent? Overall, these sequences represented 361 species belonging to 121 genera. Each individual was colonized by 98 ± 31 species. Table 1 shows the demographic, clinical, and biometric characteristics of the groups. Since this is a meta‐analysis of 4 datasets, we tested the data for potential batch effects during sample processing and sequencing. The distribution of males and females was not significantly different between the 4 datasets (*p >* 0.05, ANOVA, however, 2 of the datasets contained more younger (<30 years) subjects (*p <* 0.05, ANOVA).

**TABLE 1:** Demographic characteristics of groups in the study.

	Female	Male	
Number of subjects	781	160	
Age range, mean (SD)	0‐77, 33 (11)	0‐81, 32 (11.3)	
Age groups: count, (mean) *p*‐values	0‐1:16, (1)	0‐1:2, (1)	*p*‐value: 0.08
	2‐5:22, (2)	2‐5:16, (2)	*p*‐value: 0.90
	6‐18:84, (12)	6‐18:62, (10)	*p*‐value: 0.17
	19‐30:98, (23)	19‐30:33, (22)	*p*‐value: 0.09
	31‐50:280, (40)	31‐50:26, (39)	*p*‐value: 0.09
	51‐65:158, (57)	51‐65:16, (57)	*p*‐value: 0.66
	65+: 123, (70)	65+:5, (72)	*p*‐value: 0.16
Ethnicity (%)	Caucasian (76.58)	Caucasian (77.30)	
	Asian (10.82)	Asian (13.83)	
	African American (7.61)	African American (2.38)	
	Middle Eastern (0.47)	Middle Eastern (2.38)	
	Hispanic (4.51)	Hispanic (2.38)	

### 3.1 Meta‐taxonomics reveals the existence of a HerBiome and a HisBiome

**FIGURE 2:**
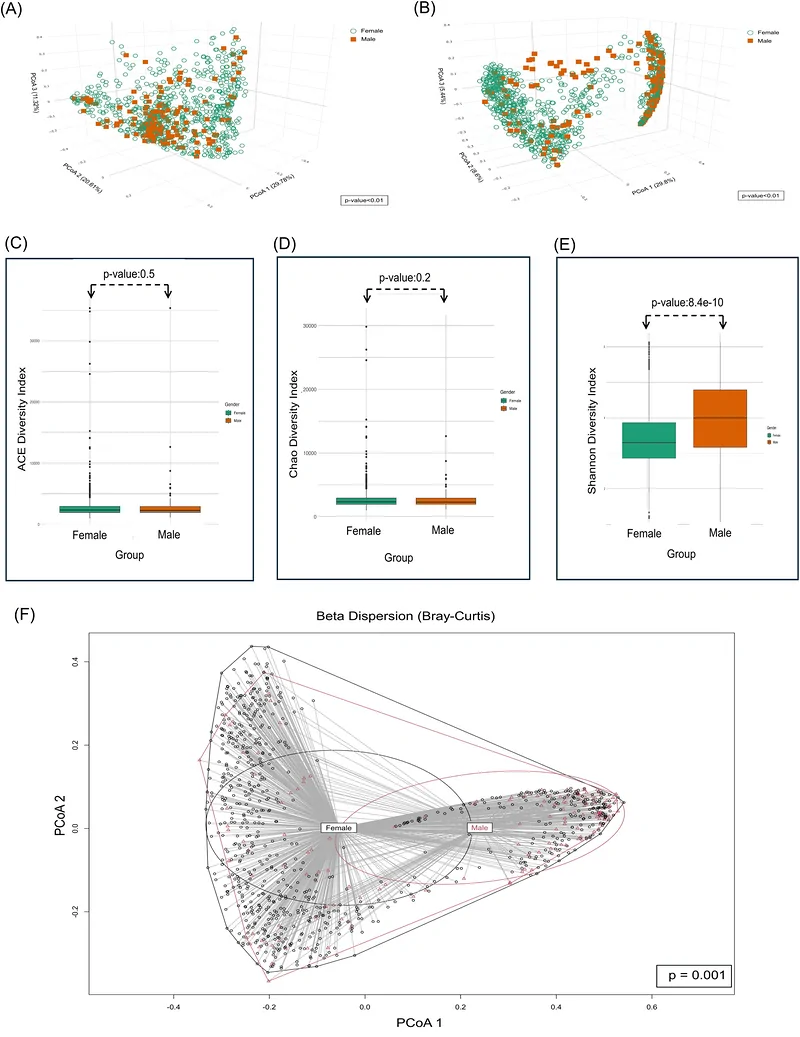
Alpha and beta diversity characteristics of the subgingival microbiome of females and males. PCA based on weighted‐UniFrac and Bray–Curtis distances (A and B, respectively) revealed statistically significant partitioning of the microbiome based on sex (*p <* 0.01, PERMANOVA). ACE (C) and Chao1 (D) diversity indices do not show significant differences (*p >* 0.05, Wilcoxon rank‐sum test) between females and males. Shannon diversity (E) was statistically different between sexes (*p =* 8.4 × 10^−10^), indicating higher microbial evenness and richness in males. Beta‐dispersion based on Bray–Curtis distance (F) was significantly greater in males than females, indicating a higher degree of microbial heterogeneity among males (*F =* 12.349, *p =* 0.001, ANOVA).

We began our analysis by estimating the similarities between the microbiomes using both branch‐length (weighted UniFrac distances) and phylotypic (Bray–Curtis Dissimilarity) metrics. PCoA of UniFrac and Bray–Curtis Indices revealed statistically significant partitioning of the microbiome based on sex (*p <* 0.01, PERMANOVA, Figure 2A,B).

We then compared the species richness and evenness between sexes using the ACE, Chao1, and Shannon Indices (Figure 2C–E). The ACE and Chao did not demonstrate significant differences between groups, indicating that males and females were colonized by the same types of species; however, a significantly higher microbial diversity was observed in males compared to females using the Shannon Index (*p =* 8.4  × 10^−10^, Wilcoxon rank‐sum test), suggesting that the male subgingival microbiome contains greater numbers of species with differing relative abundances. Beta‐dispersion of the Bray Curtis Index (distance from multivariate centroids of the group) was significantly greater in males than females, indicating a higher degree of microbial heterogeneity among males (*F =* 12.349, *p =* 0.001, ANOVA, Figure 2F). Furthermore, beta‐dispersion increased significantly with age (*F =* 22.66, *p =* 0.001), with adults (18–30 years and older) showing markedly higher community heterogeneity compared to younger individuals (see Supplementary Figure 1 in the online *Journal of Periodontology*).

**FIGURE 3:**
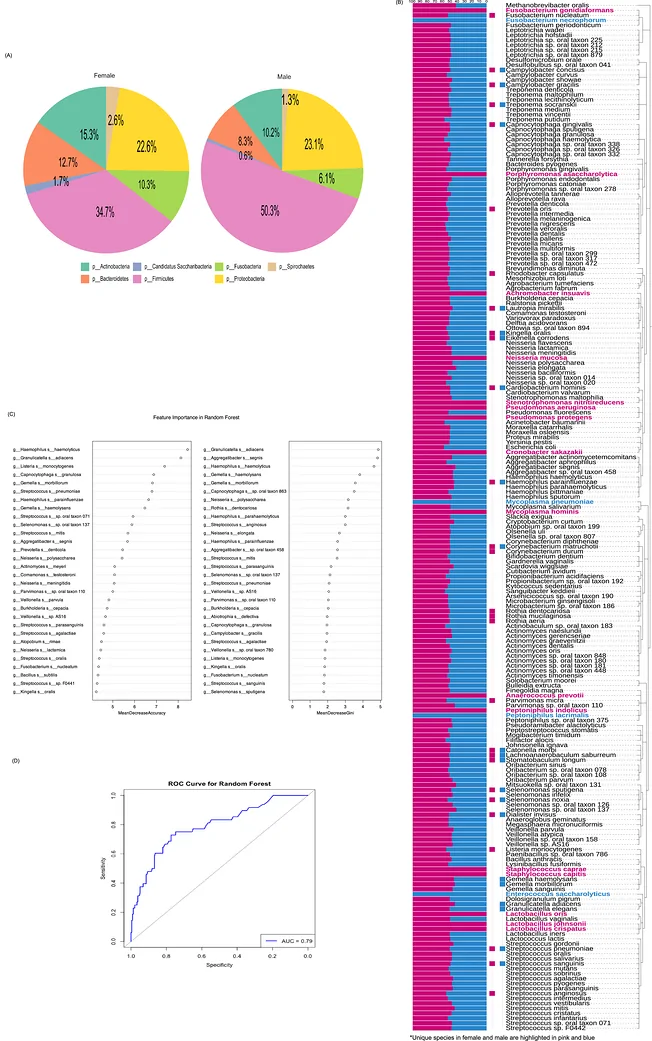
Microbial composition and machine learning‐based classification of sex‐specific profiles. (A) Phylum‐level differences between females and males demonstrate significantly higher Firmicutes in males than females (p < 0.001, Wilcoxon rank‐sum test), while Bacteroidetes, candidatus Saccharibacteria, Actinobacteria, Fusobacteria, and Spirochaetes were over‐represented in females than males (p < 0.001 Wilcoxon rank‐sum test). (B) Species‐level differences are shown. Pink and blue bars represent relative abundances (log 10 transformed) of differentially abundant taxa (p < 0.05, FDR‐adjusted Wald test, DESeq2) in females and males respectively, and the species identified in 80% or more individuals (core microbiome) in each group are represented by colored squares adjacent to taxa names. Taxa uniquely identified in either sex are highlighted by colored font. (C) The importance of these taxa in identifying sex using a machine learning algorithm. (D) ROC for this model is shown, demonstrating a classification performance (AUC) of 0.79. This figure can be enlarged in the online version of the journal.

We next conducted a comprehensive analysis to evaluate the differences in microbial community structures between sexes. At the phylum level, abundance of *Firmicutes* was significantly higher in males (50.3%) than females (34.7%) (Figure 3A, *p <* 0.001, Wilcoxon rank‐sum test). On the other hand, *Bacteroidetes*, *Candidatus Saccharibacteria*, *Actinobacteria, Fusobacteria*, and *Spirochaetes* were over‐represented in females than males (*p <* 0.001, Wilcoxon rank‐sum test). Several species, notably those belonging to the genera *Lactobacillus* and *Staphylococcus* were uniquely identified in females, while fewer species were unique to men. Among the 361 species shared by both sexes, 223 demonstrated significant differences between males and females (Figure 3B (iToL tree) *p <* 0.05, FDR‐adjusted Wald test). Notable among these were species belonging to the genus *Lactobacillus, Fusobacterium, Leptotrichia, Campylobacter*, and *Streptococcus*.

A machine learning model trained on this dataset using class weight balance was able to identify males and females with a sensitivity of 0.99, specificity of 0.15 and accuracy of 85% (Figure 3C,D). *Granulicatella adiacens* and *Gemella morbillorum* emerged the most influential features. Species belonging to the genera *Haemophilus*, *Streptococcus*, and *Neisseria* also demonstrated strong discriminatory power.

### 3.2 The subgingival microbiome demonstrates decreasing diversity over the human healthspan

**FIGURE 4:**
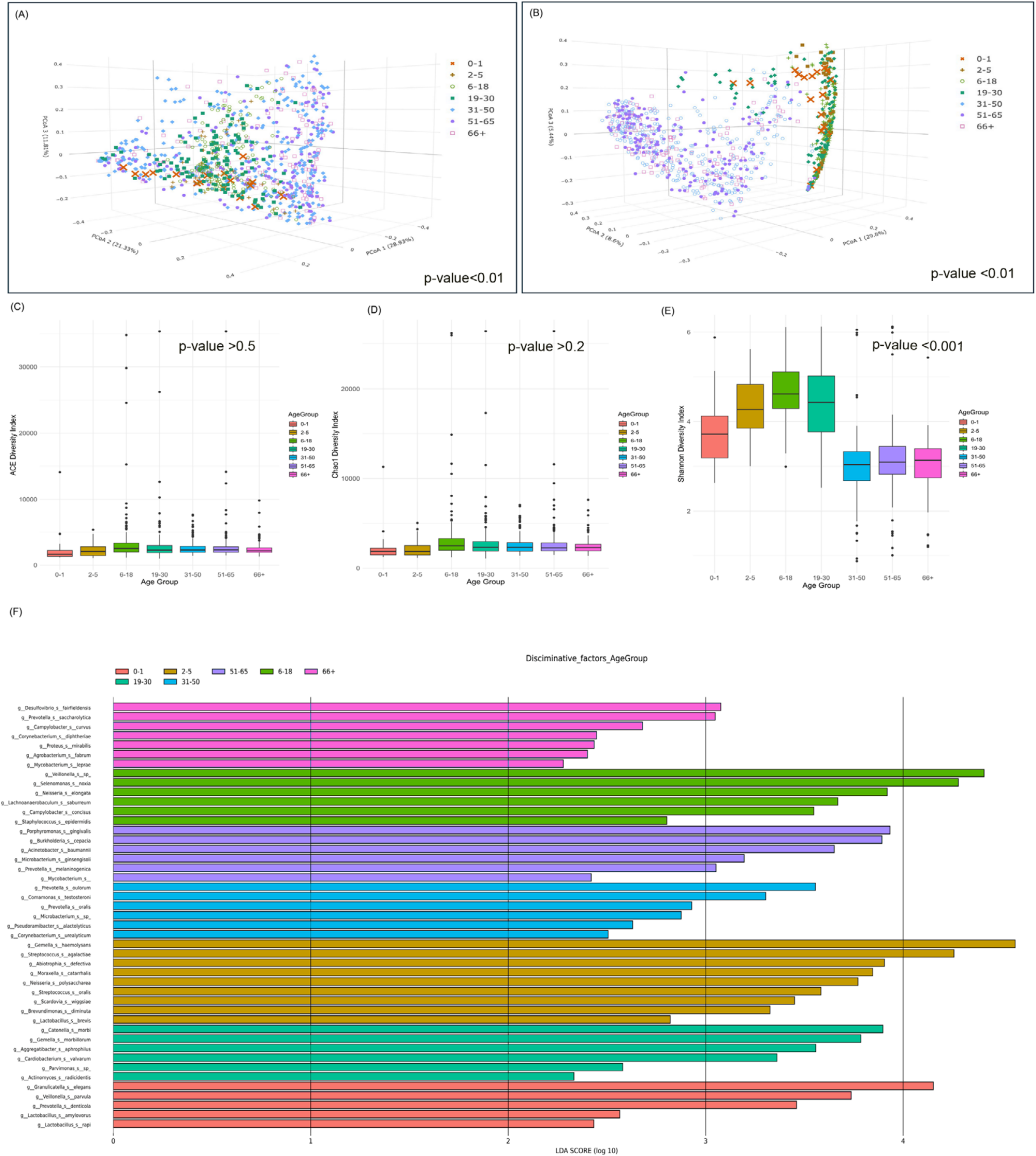
Alpha and beta diversity characteristics of the subgingival microbiome based on age. PCA based on weighted‐UniFrac and Bray–Curtis distances (A and B, respectively) revealed statistically significant partitioning of the microbiome based on sex (p < 0.01, PERMANOVA). ACE (C) and Chao1 (D) diversity indices do not show significant differences (p > 0.05, Wilcoxon rank‐sum test) between females and males. (E) Shannon diversity was statistically different between sexes (p < 0.01), indicating higher microbial evenness and richness in males. (F) LEfSe (Linear Driscriminant Analysis Effect Size) bar plot of the taxonomical differences in different age groups is shown. Each bar represents the effect size (LDA) score for each taxa in a certain group. The length of each bar represents the log10 transformed LDA score.

We next investigated the association between age and the subgingival microbiome by replicating the same analyses as above, except with age as the independent variable. PCA of UniFrac and Bray–Curtis distances revealed statistically significant partitioning of the microbiome based on age (*p <* 0.01, PERMANOVA, Figure 4A,B). ACE and Chao did not demonstrate significant differences between different age groups; however, a significantly higher Shannon Index was observed in younger (age < 30) compared to older individuals (*p <* 0.001, Wilcoxon rank‐sum test, Figure 4C–E). LefSe identified taxonomical differences in microbial composition over the human lifespan, with younger age groups (e.g., 0–1 and 2–5, in coral and golden, respectively) showing distinct bacterial profiles compared to middle‐aged (45–40) and elderly (> 65) individuals (Figure 4F). Notably, taxa such as *Desulfovibrio fairfieldensis*, *Prevotella saccharolytica*, and *Campylobacter curvus* were prominent in the 66+ age group, whereas *Streptococcus salivarius* and *Gemella haemolysans* were enriched in the 2–5 age group.

### 3.3 Mediation analysis reveals that age is an important determinant of the HerBiome

**FIGURE 5:**
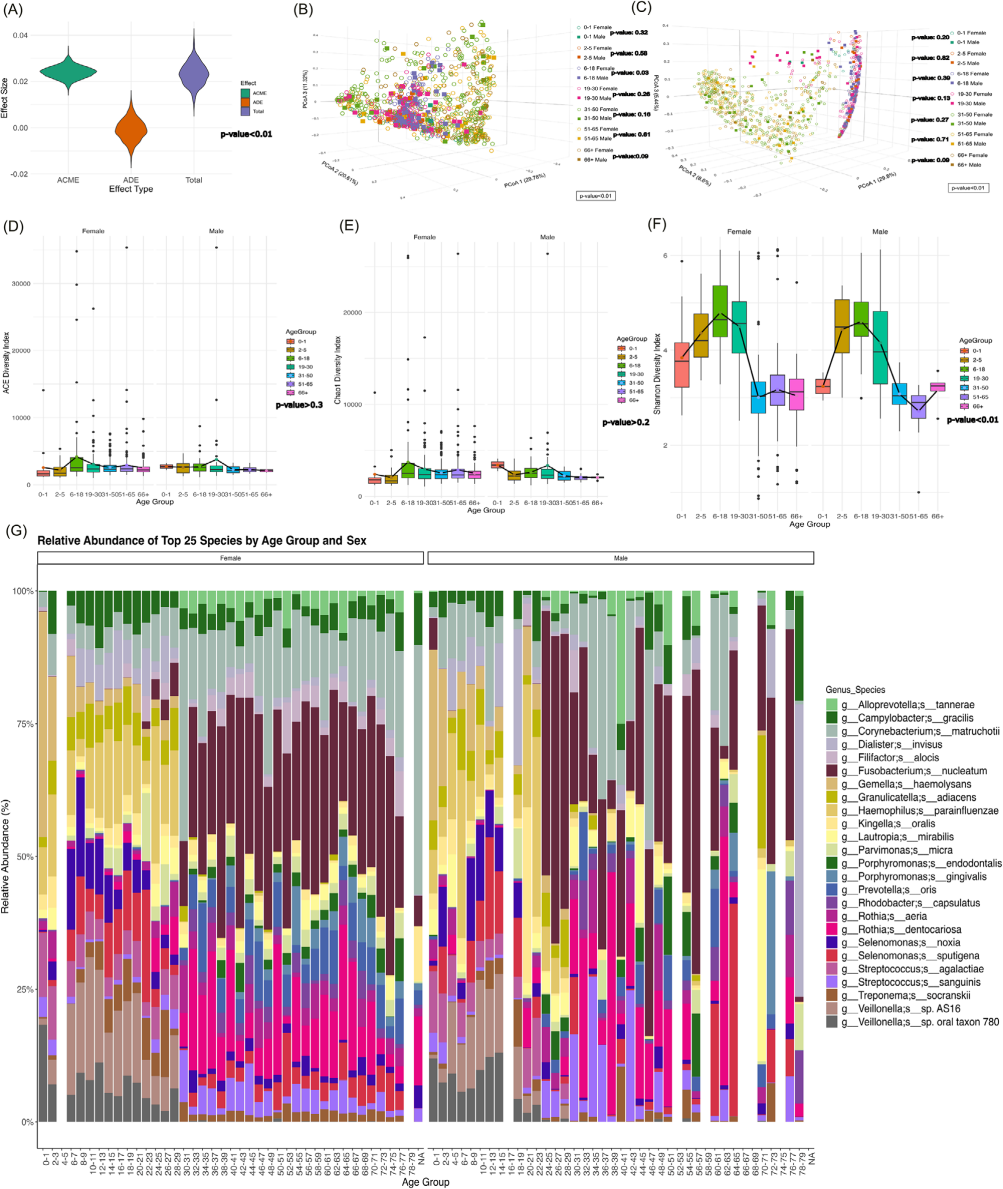
Alpha and beta diversity characteristics of the subgingival microbiome based on sex and age. (A) Mediation analysis to assess the influence of age as a modifier of sex differences is shown. ADE was −0.000964, while the ACME was 0.024052, which was almost identical to the total effect of 0.023088 (p < 0.01), indicating that age exerts an almost complete mediation effect. PCA plots illustrating beta diversity of the microbial communities based on sex and age using weighted UniFrac distances (B) and Bray–Curtis dissimilarity (C). Each point represents a sample, colored according to age group and sex. (D–F) The alpha diversity based on age groups and sex for ACE, Chao1, and Shannon Diversity Index. Relative abundances of top 25 species in females and males are shown in Panel G with respect to age groups.

To further investigate whether the subgingival sexome is influenced by age, we deconstructed sex as an explanatory variable of microbial diversity with age as a mediator. The average direct effect (ADE) was −0.000964, while the average causal mediation effect (ACME) was 0.024052, which was almost identical to the total effect of 0.023088 (*p <* 0.01, Figure 5A), indicating that age exerts an almost complete mediation effect. In support of this, PCoA of UniFrac and Bray–Curtis distances demonstrated significant clustering of microbiomes based on both age and sex (*p <* 0.01, PERMANOVA, Figure 5B,C). Notably, sex‐specific differences in beta‐diversity were age dependent: female and male microbiomes differed significantly within the 6–18‐year age group using the UniFrac metric (*p =* 0.03), while a trend toward sex‐associated divergence was observed in individuals aged ≥66 years (*p =* 0.09). In further corroboration, the Shannon index was significantly lower in females >31 years of age when compared to younger females. Such a difference was not evident in males. By contrast, ACE and Chao indices did not demonstrate differences over time in either sex (Figure 5D,F). Phylum‐level analysis revealed that in females, *Firmicutes* were notably dominant during early life (0–25), contributing to a large proportion of the microbial community, with a sharp decline in this phylum around age 29. There were concomitantly higher levels of phyla such as *Proteobacteria, Bacteroidetes*. By contrast, males demonstrated persistent instability in phyla distribution, with no clear stabilization phase, suggesting possible sex‐specific microbiome regulation mechanisms (Supplementary Figure 2 in the online *Journal of Periodontology*). When the data were deconvoluted to the species level, the most notable observation was a significant enrichment in *Fusobacterim nucleatum, Rothia aeria*, and *Rothia dentocariosa*, with a concomitant depletion in abundances *Selenomonas noxia, Veillonella* sp. AS16 and *Veillonella* oral taxon 780 at 31 years of age in females, while no specific species was associated with age in men (Figure 5G).

## 4 DISCUSSION

The impact of age and sex on the subgingival microbiome have been separately studied, however, the lifelong changes in these ecosystems have not been explored in depth. Utilizing high‐throughput sequencing, our investigation focused on uncovering sex‐specific differences in microbiome composition, identifying the age at which these differences become apparent, determining species responsible for these discrepancies, and understanding how age influences microbial variations.

We have previously demonstrated that the subgingival microbiome is influenced by dentition state,^23^ ethnicity,^38^ smoking,^39^ vaping,^40^ diabetes,^41^ rheumatoid arthritis^42^ and pregnancy.^43^ Therefore, we carefully selected individuals with 20 or more teeth (except in pre‐dentate infants and some children in primary dentition), who were non‐diabetic, never‐smokers, non‐vapers and not pregnant at the time of sample collection.

Our key finding is that the trajectory and pattern of aging of the subgingival microbiome differs between males and females. Two lines of evidence support this finding. The first is that in females, age is a facilitator of microbiome changes, and that early life is a period of dynamic microbiome fluctuations that stabilizes after 30 years. Such a defined trajectory could not be discerned in males. Consistent with this age‐dependent effect, sex‐associated differences in beta‐diversity were not uniform across the lifespan: female and male microbiomes differed significantly within the 6–18‐year age group based on UniFrac distances, while a trend toward divergence was observed in individuals aged ≥66 years, suggesting that sex effects on community composition are most pronounced during periods of biological transition. The second is that males demonstrated a greater beta‐dispersion when compared to females at all age decades, pointing to a microbiome that is personalized to each individual. Evidence emerging from several animal (vole, lizard etc.)^44^, ^45^ and environmental (coral reefs, bivalves etc.)^46^, ^47^ microbiomes suggests that personalization signifies the inability of either the host or microbial ecosystem to regulate community membership and/or relative abundances of species; leading to the creation of dysbiotic communities in response to stressors. This concept is known as the Anna Karenina Principle,^48^ following on Leo Tolstoy's observation that “all happy families look alike; each unhappy family is unhappy in its own way”. We have previously observed this effect in periodontitis‐associated microbiomes, which demonstrate a high degree of heterogeneity;^49^ and it is interesting to note this pattern in periodontally and systemically healthy males. This finding might have important implications for the higher prevalence of periodontitis observed in males.^7^, ^8^, ^9^, ^50^

Even more importantly, our data suggest why older males might have an increased susceptibility to periodontitis. Beta‐dispersion was significantly higher in aged (> 65) when compared to young (< 35) and middle aged (45–60) males, pointing to an increasing personalization in the male microbiome with age. Dysbiosis has been identified as one of the 12 hallmarks of aging,^51^ and studies from the gut demonstrate that aging is associated with an increase in personalization.^52^ It is acknowledged that inflammaging, immunosenescence, changes in the structure and function of the periodontal tissues can contribute to increased susceptibility to periodontitis^53^, ^54^; our data suggest that aging‐associated dysbiosis could be an additional factor, especially in males.

One of the most interesting findings was the dramatically higher abundance of the oral pathobiont *Fusobacterium nucleatum* in females > 30 years of age. Our findings are startlingly similar to a previous study using cloning and sequencing of the 16S–23S ITS region on 60 females from eleven countries in 5 continents;^55^ arguing against this being an artifact of sequence and/or data analysis. *F. nucleatum* is an ubiquitous species in the oral microbiome^56^, and is known to mediate adhesion by tertiary colonizers (bridge species).^57^ While it has long been implicated in adverse pregnancy outcomes,^58^, ^59^, ^60^ evidence is emerging regarding its role endometriosis^61^ as well as colorectal cancer.^62^

Studies on the gut microbiome demonstrate that a sharp reduction in diversity underlies the pathogenesis of gastro‐intestinal disease.^63^ Taken together, the high abundance of *F. nucleatum* coupled with sharply reduced alpha diversity in women over 30 suggests a higher propensity for oral diseases to impact other organ systems in females. Further studies are warranted on the link between oral diseases and overall health, with a special focus on females.

In summary, within the limits of a cross‐sectional analysis, and metataxonomic characterization of the microbiome, we find evidence to support the hypothesis that sex in an important determinant of the subgingival microbiome. Not only are males and females microbially different at most age decades, but also their aging trajectories and pattern vary widely. However, it is important to note that the cross‐sectional nature of our study precludes us from directly assessing within‐person microbiome changes across the lifespan. The observed differences in diversity and composition between age groups may reflect true aging trajectories but could also be influenced by cohort effects or other unmeasured group‐level variables. As such, our findings should be considered hypothesis‐generating, and longitudinal studies—sampling the same individuals over time—are needed to determine the real dynamics and causal directions of these associations. These findings have important implications for the etiopathogenesis of periodontitis in either sex, as well as the potential to personalize therapy based on age and sex. Further studies using more comprehensive ‐omics approaches are needed to unravel the specifics of these differences and their implications for resilience and resistance to future disease.

## AUTHOR CONTRIBUTIONS

**Kazune Pax**: Sample acquisition; periodontal evaluation; data analysis; and manuscript preparation. **Michelle Beverly**: Patient recruitment and consent; periodontal evaluation; sample and data collection; and manuscript preparation. **Rahul Nikam**: Sequence analysis; data analysis; and manuscript preparation. **Purnima Kumar**: Study design; sample processing; sequence analysis; data analysis; and manuscript preparation.

## CONFLICT OF INTEREST STATEMENT

None of the authors have any conflicts to report with respect to the present study.

## Supporting information



Supporting Information

Supporting Information
