# Supplementary material for: Mapping the subgingival HerBiome and HisBiome over the human healthspan
Source: J Periodontol. 2026 Mar 23;97(8):1659–71. doi: 10.1002/jper.70098 (PMC13502437; doi:10.1002/jper.70098)
Supplement: Supplementary file 1 [file JPER-97-1659-s001.pdf]

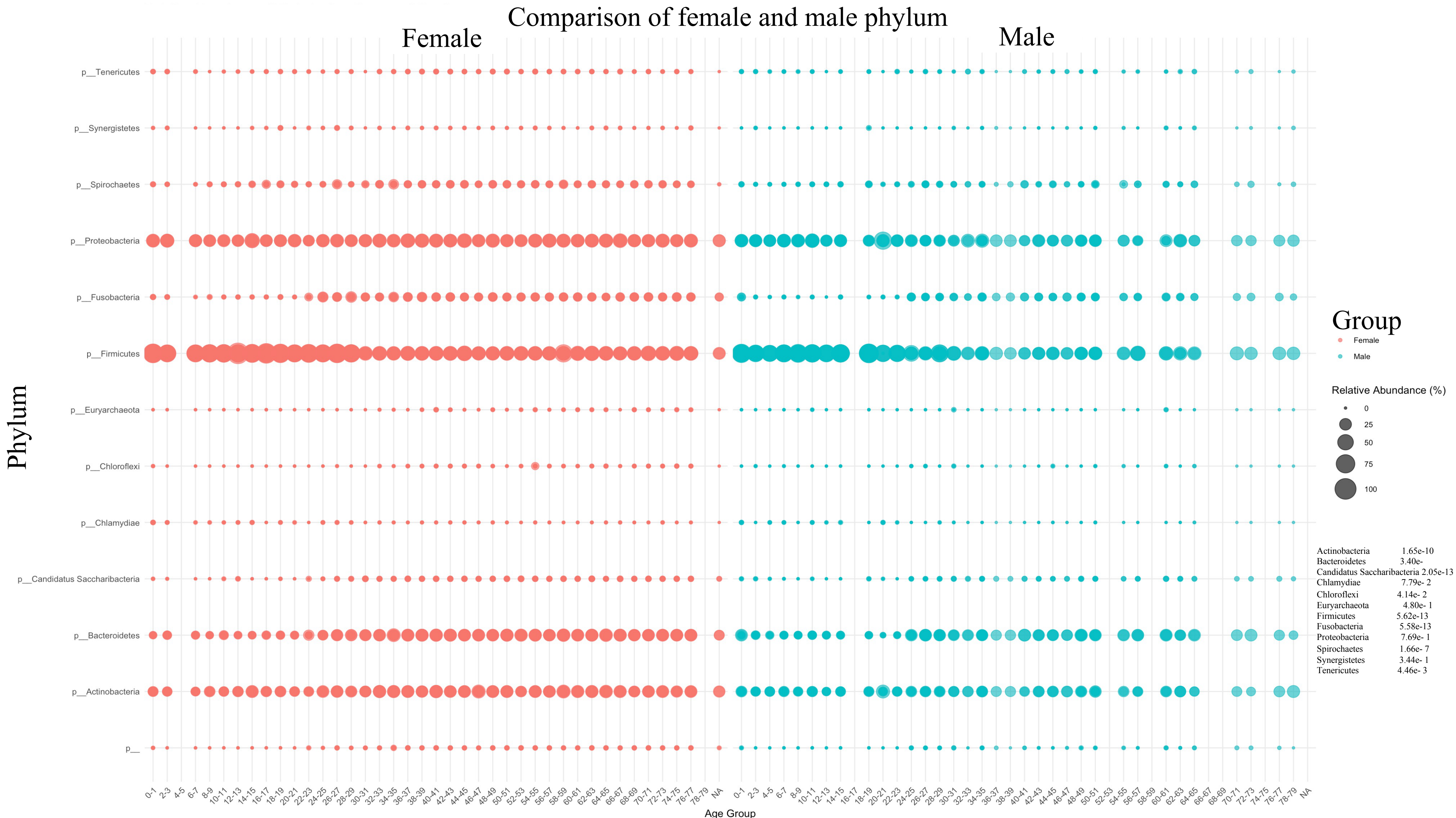
Comparison of female and male phylum
Female
Male
Phylum
p__Tenericutes
p__Synergistetes
p__Spirochaetes
p__Proteobacteria
p__Fusobacteria
p__Firmicutes
p__Euryarchaeota
p__Chloroflexi
p__Chlamydiae
p__Candidatus Saccharibacteria
p__Bacteroidetes
p__Actinobacteria
p__
Age Group
Group
Female
Male
Relative Abundance (%)
0
25
50
75
100
Actinobacteria 1.65e-10
Bacteroidetes 3.40e-
Candidatus Saccharibacteria 2.05e-13
Chlamydiae 7.79e- 2
Chloroflexi 4.14e- 2
Euryarchaeota 4.80e- 1
Firmicutes 5.62e-13
Fusobacteria 5.58e-13
Proteobacteria 7.69e- 1
Spirochaetes 1.66e- 7
Synergistetes 3.44e- 1
Tenericutes 4.46e- 3
