# Supplementary material for: Mapping the subgingival HerBiome and HisBiome over the human healthspan
Source: J Periodontol. 2026 Mar 23;97(8):1659–71. doi: 10.1002/jper.70098 (PMC13502437; doi:10.1002/jper.70098)
Supplement: Supplementary file 2 [file JPER-97-1659-s002.pdf]

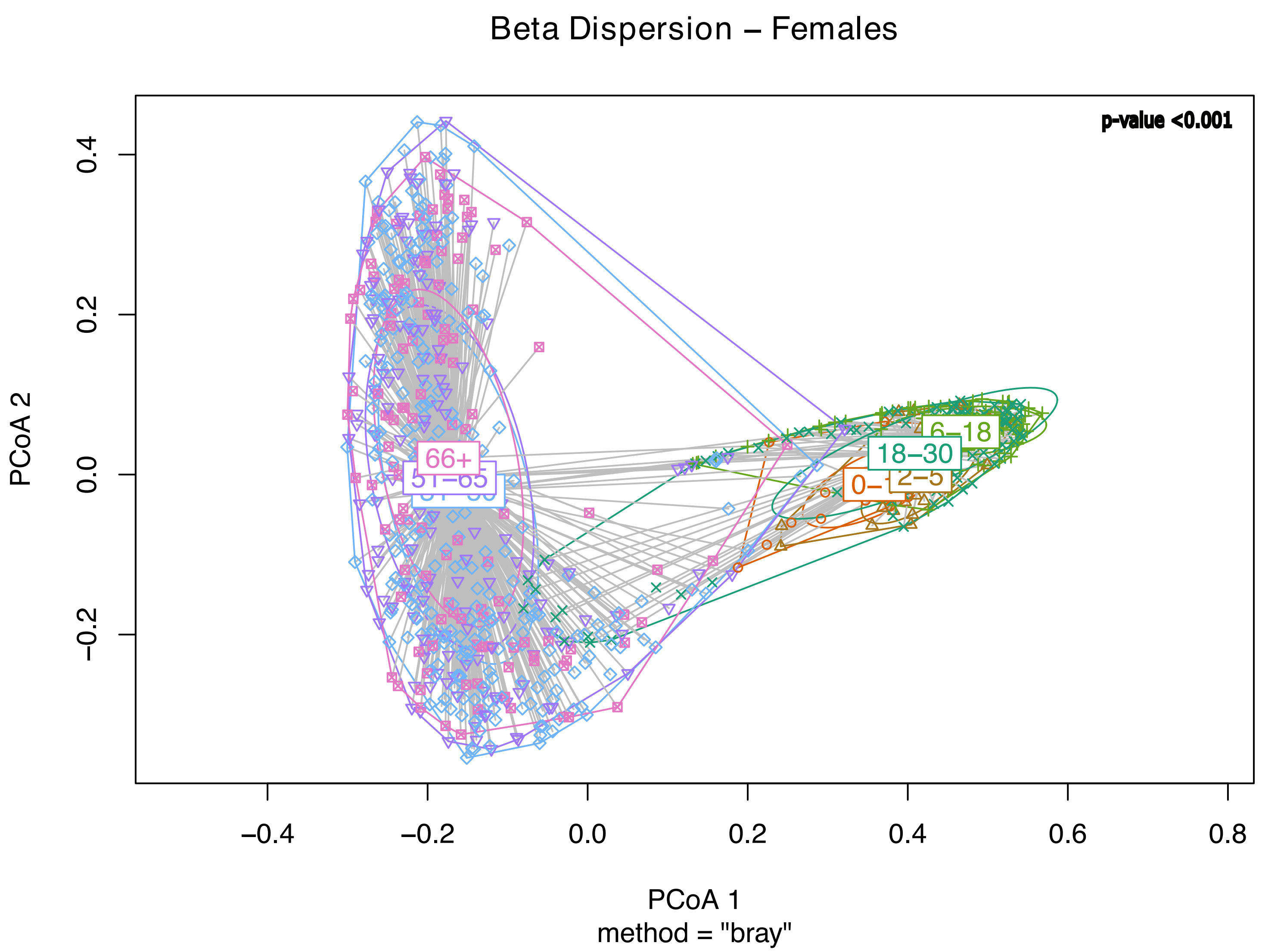

Beta Dispersion – Females
p-value <0.001
66+
51–65
6–18
18–30
0–1
2–5
PCoA 1
method = "bray"
PCoA 2

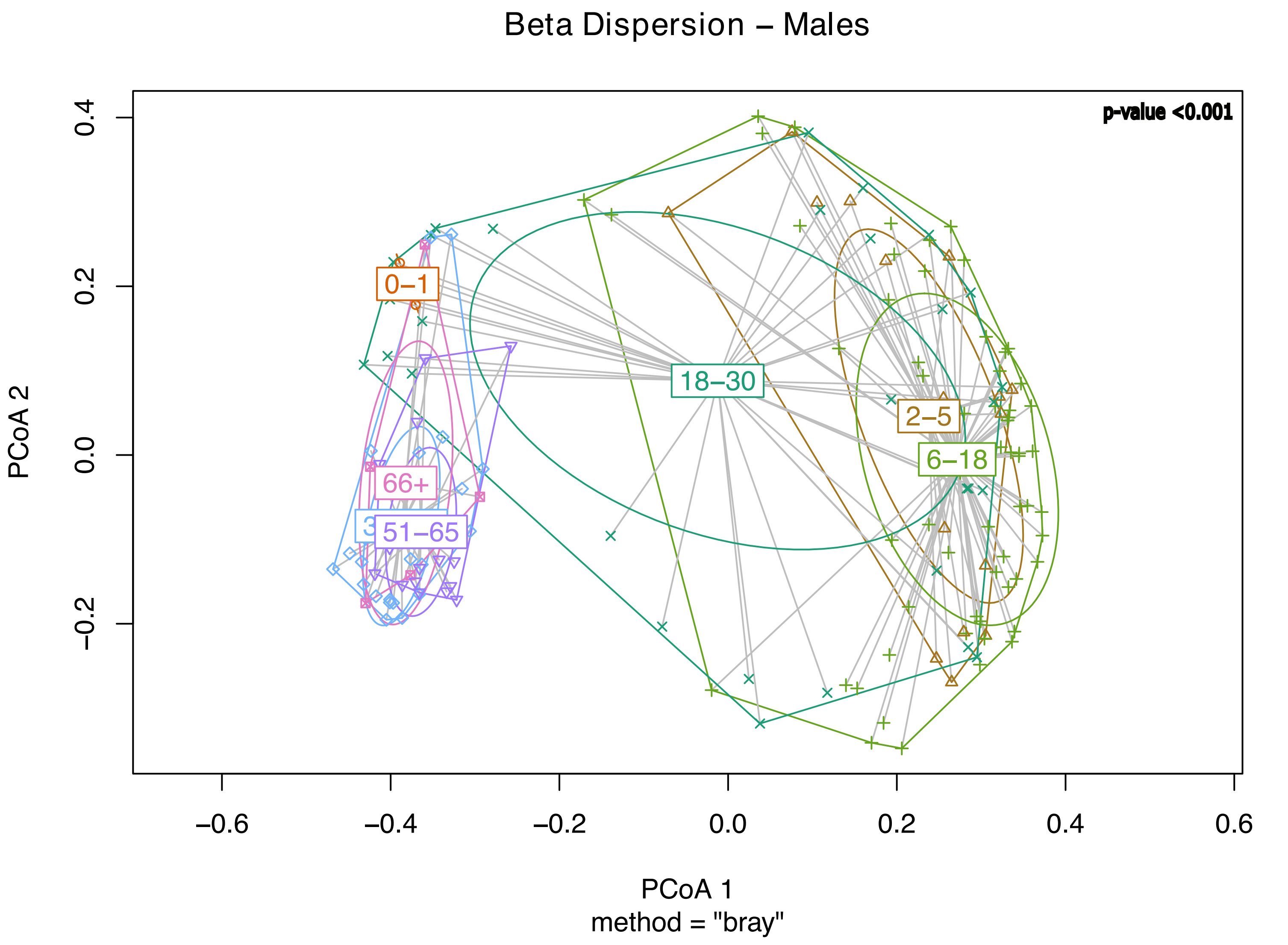

Beta Dispersion – Males
p-value <0.001
0–1
18–30
2–5
6–18
66+
51–65
PCoA 1
method = "bray"
PCoA 2
